# Evaluation of a Localized Treatment Technique Using Three Ready-to-Use Products Against the Drywood Termite *Incisitermes snyderi* (Kalotermitidae) in Naturally Infested Lumber

**DOI:** 10.3390/insects3010025

**Published:** 2012-01-03

**Authors:** Robert Hickman, Brian T. Forschler

**Affiliations:** 1 BASF Corporation, Pest Control Solutions, 3568 Tree Court Ind. Blvd., St. Louis, MO 63122, USA; E-Mail: robert.hickman@basf.com; 2 Department of Entomology, University of Georgia, Athens, GA 30602, USA

**Keywords:** isoptera, management, termite detection device

## Abstract

Twenty-one boards infested with drywood termites were examined for activity using a Termatrac® motion detector. Termite galleries were identified using a Resistograph drill and treated with one of three ready-to-use (RTU) products. Results indicated that the Termatrac was excellent at locating termite activity but provided 9.5% false negatives. The Resistograph located termite galleries with an average of 4.6 ± 2.7 holes drilled to find at least one gallery in a board. Treatments included three formulations and two active ingredients; a foam (imidacloprid), a dry (fipronil) and an experimental formulation in a pressurized can (fipronil). All treatments provided evidence for a reduction in mean termite populations per board compared to the control. Two treatments provided evidence of elimination of infestation but no formulation eliminated infestations in every board that was treated. The concept of local treatment for drywood termite control is discussed relative to our results.

## 1. Introduction

Termites from the family Kalotermitidae, the drywood termites, have a worldwide distribution and can be serious structural pests in tropical to temperate coastal regions [1,2,3]. The biology of drywood termites is considered homogenous although only a few species within the family have been examined in detail [3]. Drywood termites nest in and feed on wood and are considered single-site nesters with a dynamic caste developmental system that includes neoteny [4,5]. Nest architecture is considered complex with numerous wide galleries connected by narrow passageways [6].

Control of drywood termites in structures is complicated by their biology that includes rapid development of neotenics and nest architecture that is difficult to discern from the exterior of a piece of dimensional lumber [7,8,9,10,11]. Additional issues affecting measures of efficacy of control measures include detection and non-destructive evaluation of treatments [12,13]. We conducted a study using cypress lumber naturally infested by *Incisitermes snyderi* (Light) to evaluate two questions relative to drywood termite management. First, how effective is microwave motion detection (Termatrac®) in identifying live drywood termite activity. Second, what is the degree of efficacy of three ready-to-use (RTU) formulations including an imidacloprid foam, a dry fipronil formulation applied with a compression bulb applicator and a BASF experimental formulation in a pressurized can. Results are discussed in regard to increasing the reliability of and level of confidence in drywood termite local or spot treatments.

## 2. Materials and Methods

Termites. Naturally infested cypress (Cupressaceae) boards were collected in 2009 near a house at the University of Georgia Marine Institute on Sapelo Island, Georgia, USA. Infested boards (N = 30) were relocated to another, more isolated, area on the island and rearranged in two stacks by placement on cinder blocks at least 20-cm above soil level. The cypress boards used in this 2010 study were custom cut and measured 2.6 × 15.8-cm × 3.3-m (D:W:L).

Detection of termite activity. Boards were numbered and a tape measure placed lengthwise down the middle of each board. On either side of the tape measure, readings were taken every 15 cm creating 22 separate sections for recording data. The site for each section therefore remained consistent throughout the study. Boards were placed, one at a time, on a platform supporting both ends approximately 0.6 m above the ground. Termite activity was measured using a Termatrac® (65 Christensen RoadStapylton, 4207 Queensland, Australia) microwave motion detector by placing it on each of the 22 sections for at least 15 seconds. Indications of motion, as per Termatrac® instructions, assumed to be drywood termite activity, were recorded by board number and board section. Activity was recorded one day before treatment (August 23, 2010), the day of treatment (August 24, 2010), and 65 days after treatment (October 28, 2010). Activity data were compared for each board by number of active sites per board and location of sites within boards.

Experimental Design. The activity data, by board, was used to place individual boards into one of 5 categories based on number of active sections—Category I; 1-2 active sections, Category II; 4-5, Category III; 7-9, Category IV; 10-11, and Category V; ≥13 (Table 1 & Table 5). Boards were then randomly assigned to treatments based on Category classification to equilibrate pre-treatment measures of activity across treatments assuming Termatrac® activity measures correlated with number of termites/level of infestation.

**Table 1:** Number of active sections by board as indicated by Termatrac readings on three separate days and the number of termites, by caste, found per board 65 days after treatment.

Board #	Treatment	# active	# active	# active	#	#	#
		Pre-trt	Day of	65 days	65 days	65 days	65 days post
	**control**						
12	IV	10	7	3	715	45	2
10	I	1	1	1	6	1	1
18	III	7	7	11	1113	23	8
9	IV	11	11	10	1143	23	4
22	V	14	16	15	1039	41	2
	**foam**						
14	I	1	2	1	25	0	0
20	V	13	12	4	622	12	4
26	II	5	6	5	157	9	5
15	IV	10	10	8	1319	40	4
7	III	7	6	3	433	9	2
	**dust**						
25	II	4	8	2	157	3	2
16	IV	10	8	3	335	6	4
6	I	1	2	0	36	0	2
1	V	13	9	0	0	0	0
19	I	2	3	0	0	0	0
11	III	7	3	0	0	0	0
	**exp**						
13	III	8	9	0	0	0	0
17	III	9	8	3	52	2	0
3	II	4	2	0	0	0	0
21	IV	11	10	1	229	0	2
2	V	16	8	0	5	1	0

Twenty-one boards were selected for treatment, treated and restacked outdoors by treatment regime. Individual boards, within treatment, were separated to prevent movement of termites between boards by using 2, 3 × 3 × 10-cm wood blocks covered in aluminum foil placed 40-cm from the two ends of each board. 

Treatments. Immediately prior to treatment a resistograph drill (IML-Instrumenta Mechanic Labor GmbH, 1275 Shiloh Road, Ste. 2780–30144 Kennesaw, GA, USA) was used to locate at least one gallery at two locations (61-cm mark and 122-cm mark) regardless of the site of activity in that board. Resistograph drill holes were placed within 5-mm of the 61-mm or 122-cm mark on the edge of a board and drilled through the length of the board at various heights relative to the 2.6-cm width. The resistograph drill hole that indicated at least one gallery at that respective location was used as the point of application of the appropriate treatment.

**Table 2:** Mean number of galleries per treatment location in boards by treatment type as determined by resistograph readings and number of resistograph drill attempts per board required to find at least one gallery for treatment.

Treatment	Mean number of galleries per board		
	@ 61-cm	@ 122-cm	Resistograph holes/board
Control	2.4 ± 0.5	2.2 ± 0.4	3.2 ± 2.4
Foam	1.8 ± 0.8	2.8 ± 0.8	4.0 ± 1.4
Dust	1.5 ± 0.5	1.3 ± 1.3	6.8 ± 2.9
EXP	2.2 ± 1	2.2 ± 1.2	3.8 ± 1.7

**Table 3:** Mean number (±SD) of active sections per board by treatment as determined by Termatrac® motion detector.

Treatment	Mean number of active sections per board		
	One day prior	Day of treatment	65 days post treatment
Control	8.6 ± 4.4	8.4 ± 5.5	8 ± 5.2
Foam	7.2 ± 4.1	7.2 ± 3.5	4.2 ± 2.3
Dust	6.2 ± 4.3	5.5 ± 2.9	0.8 ± 1.2
EXP	9.6 ± 3.9	7.4 ± 2.8	0.8 ± 1.2

**Table 4:** Mean number of termites per board by treatment, caste, and the number of boards where no termites were found 65 days after treatment by destructive sampling.

**Treatment and number of boards per treatment**	**Mean number of termites per board**			**Number of boards with no termites**
	Workers	Soldiers	Reproductives	
Control N = 5	803 ± 427	27 ± 16	3 ± 2	0
Foam N = 5	511 ± 454	14 ± 14	3 ± 2	0
Dust N = 6	88 ± 124	1 ± 2	1 ± 1	3
EXP N = 5	57 ± 88	0.6 ± 0.8	0.4 ± 0.8	2

There were four treatments. The 0.05% imidacloprid foam, designated as ‘foam’ in discussion of results, was applied until an excessive amount of foam leaked from other holes in the board. This generally took 1–3 seconds before excessive leakage occurred resulting in approximately 6.5 mL–19.5 mL being applied (assuming 26 mL/4 s) per hole.

The 0.5% fipronil dry formulation, designated as ‘dry’ in discussion of results, was applied using three compressions from a proprietary compression bulb applicator. This application resulted in approximately 0.1 grams of formulation applied to each hole.

The experimental formulation, designated as ‘exp’ in discussion of results, was applied from a pressurized can at the rate of depressing the applicator valve for one second per hole.

Control boards received an application of the ‘dry formulation’ (no active ingredient) as per directions described for the 0.5% fipronil dry formulation treatment.

**Table 5:** Table illustrative of the relative position (left or right-side of board), by board section and date, of termite activity as measured using the Termatrac® listed by treatment including pre-treatment activity category of the board and the number of galleries identified for treatment by position for each board as measured using the Resistograph.

Control	Date of Termatrac® measurement and activity			Number of galleries treated by location
BOARD # 12 Category IV	Pre-treatment	Day of treatment	Post-treatment evaluation	
15-cm				
30-cm	X	X		
46-cm				
61-cm	X	X	X	2
76-cm	X	X	X	
91-cm	X		X	
106-cm	X	X		
122-cm	X	X		2
137-cm	X X	X X		
152-cm	X			
167-cm				
**Control**				
**BOARD # 10** ** Category I**	**Pre-treatment**	**Day of treatment**	**Post-treatment evaluation**	
15-cm				
30-cm				
46-cm				
61-cm				2
76-cm				
91-cm		X		
106-cm	X		X	
122-cm				2
137-cm				
152-cm				
167-cm				
**BOARD # 18 Category III**	**Pre-treatment**	**Day of treatment**	**Post-treatment evaluation**	
15-cm			X	
30-cm	X	X X	X X	
46-cm	X X	X X	X X	
61-cm	X	X	X X	3
76-cm	X	X	X X	
91-cm	X	X		
106-cm	X		X	
122-cm				2
137-cm				
152-cm				
167-cm			X	
**BOARD # 9** ** Category IV**	**Pre-treatment**	**Day of treatment**	**Post-treatment evaluation**	
15-cm		X	X	
30-cm	X X	X X	X X	
46-cm	X X	X	X X	
61-cm	X X	X X	X	3
76-cm	X	X	X	
91-cm	X	X	X	
106-cm	X	X	X	
122-cm	X	X		2
137-cm				
152-cm			X	
167-cm			X	
**Control**				
**BOARD # 22 Category V**	**Pre-treatment**	**Day of treatment**	**Post-treatment evaluation**	
15-cm	X	X	X X	
30-cm	X X	X	X X	
46-cm	X X	X X	X X	
61-cm	X X	X X	X X	2
76-cm	X	X X	X	
91-cm	X X	X X	X X	
106-cm	X	X X	X	
122-cm	X	X	X	2
137-cm	X	X	X	
152-cm	X	X	X	
167-cm		X		
**Foam**				
**BOARD # 14** ** Category I**	**Pre-treatment**	**Day of treatment**	**Post-treatment evaluation**	
15-cm				
30-cm				
46-cm				
61-cm				0
76-cm				
91-cm				
106-cm				
122-cm	X	X		2
137-cm		X	X	
152-cm				
167-cm				
**Foam**	**Date of Termatrac** **® measurement and activity**			**Number of galleries treated by location**
**BOARD # 20 Category V**	**Pre-treatment**	**Day of treatment**	**Post-treatment evaluation**	
15-cm		X		
30-cm	X X	X		
46-cm	X X	X	X	
61-cm	X	X		2
76-cm	X	X		
91-cm	X	X		
106-cm	X	X	X	
122-cm	X	X		3
137-cm	X X	X X		
152-cm	X	X	X	
167-cm		X	X	
**Foam**				
**BOARD # 26** ** Category II**	**Pre-treatment**	**Day of treatment**	**Post-treatment evaluation**	
15-cm		X	X	
30-cm		X	X	
46-cm	X	X	X	
61-cm	X X	X		2
76-cm	X		X	
91-cm	X	X		
106-cm				
122-cm		X	X	3
137-cm				
152-cm				
167-cm				
**Foam**				
**BOARD # 15 Category IV**	**Pre-treatment**	**Day of treatment**	**Post-treatment evaluation**	
15-cm				
30-cm	X			
46-cm	X	X	X	
61-cm	X	X		2
76-cm	X	X	X	
91-cm	X	X	X	
106-cm	X	X	X	
122-cm	X	X	X	4
137-cm	X	X	X	
152-cm	X	X	X	
167-cm	X	X X	X	
**BOARD # 7** ** Category III**	**Pre-treatment**	**Day of treatment**	**Post-treatment evaluation**	
15-cm				
30-cm				
46-cm	X	X		
61-cm	X	X	X	3
76-cm	X	X	X	
91-cm	X	X	X	
106-cm	X	X		
122-cm	X	X		2
137-cm	X			
152-cm				
167-cm				
**Dust**				
**BOARD # 25 Category II**	**Pre-treatment**	**Day of treatment**	**Post-treatment evaluation**	
15-cm		X		
30-cm	X	X		
46-cm	X	X		
61-cm		X		1
76-cm				
91-cm		X		
106-cm				
122-cm		X		1
137-cm				
152-cm			X	
167-cm	X X		X	
**Dust**				
**BOARD # 16** ** Category IV**	**Pre-treatment**	**Day of treatment**	**Post-treatment evaluation**	
15-cm		X		
30-cm				
46-cm				
61-cm	X X			2
76-cm	X	X		
91-cm	X	X		
106-cm	X			
122-cm	X			4
137-cm	X X	X		
152-cm	X	X X	X X	
167-cm	X	X X	X	
**Dust**	**Date of Termatrac** **® measurement and activity**			**Number of galleries treated by location**
**BOARD #6 Category I**	**Pre-treatment**	**Day of treatment**	**Post-treatment evaluation**	
15-cm				
30-cm	X	X		
46-cm		X		
61-cm				2
76-cm				
91-cm				
106-cm				
122-cm			^*	0
137-cm				
152-cm				
167-cm				
**Dust**				
**BOARD # 1** ** Category V**	**Pre-treatment**	**Day of treatment**	**Post-treatment evaluation**	
15-cm		X		
30-cm				
46-cm	X			
61-cm	X X			2
76-cm	X	X		
91-cm	X	X		
106-cm	X	X		
122-cm	X	X		1
137-cm	X X	X X		
152-cm	X X	X		
167-cm	X X	X X		
**Dust**				
**BOARD # 19 Category I**	**Pre-treatment**	**Day of treatment**	**Post-treatment evaluation**	
15-cm				
30-cm				
46-cm	X			
61-cm		X		1
76-cm		X		
91-cm				
106-cm				
122-cm	X	X		1
137-cm				
152-cm				
167-cm				
**BOARD # 11** ** Category III**	**Pre-treatment**	**Day of treatment**	**Post-treatment evaluation**	
15-cm	X	X		
30-cm				
46-cm				
61-cm	X X	X		1
76-cm		X		
91-cm	X X	X		
106-cm	X X			
122-cm				1
137-cm				
152-cm				
167-cm				
**EXP**				
**BOARD # 13 Category III**	**Pre-treatment**	**Day of treatment**	**Post-treatment evaluation**	
15-cm	X	X		
30-cm	X	X		
46-cm		X X		
61-cm	X			2
76-cm	X			
91-cm		X		
106-cm	X	X		
122-cm	X	X		3
137-cm	X X	X X		
152-cm				
167-cm				
**EXP**				
**BOARD # 17** ** Category III**	**Pre-treatment**	**Day of treatment**	**Post-treatment evaluation**	
15-cm				
30-cm				
46-cm				
61-cm	X			2
76-cm	X	X	X	
91-cm	X	X	X	
106-cm	X	X		
122-cm	X	X		1
137-cm	X	X		
152-cm	X	X		
167-cm	X X	X X	X	
**EXP**	**Date of Termatrac** **® measurement and activity**			**Number of galleries treated by location**
**BOARD # 3 Category II**	**Pre-treatment**	**Day of treatment**	**Post-treatment**	
15-cm				
30-cm				
46-cm	X			
61-cm				1
76-cm				
91-cm				
106-cm		X		
122-cm	X			1
137-cm				
152-cm	X			
167-cm	X	X		
**EXP**				
**BOARD # 21** ** Category IV**	**Pre-treatment**	**Day of treatment**	**Post-treatment evaluation**	
15-cm	X	X		
30-cm	X			
46-cm	X	X		
61-cm	X	X		4
76-cm	X	X		
91-cm	X	X		
106-cm	X	X		
122-cm	X	X		2
137-cm	X	X	X **	
152-cm	X	X		
167-cm	X	X		
**BOARD # 2** ** Category V**	**Pre-treatment**	**Day of treatment**	**Post-treatment evaluation**	
15-cm	X			
30-cm			^*	
46-cm	X	X		
61-cm	X X	X X		2
76-cm	X X	X		
91-cm	X X	X X		
106-cm	X X	X		
122-cm	X X			4
137-cm	X X			
152-cm	X	X		
167-cm	X			

** indicates the section where two alate (adult) termites were found during destructive sampling, it should be noted that the seasonal swarm period is May-July for *I. snyderi*; ^* indicates the section where live termites were found during destructive sampling although no activity was noted with the Termatrac® device.

Evaluation of Treatment Efficacy. Boards were evaluated using the Termatrac®, as previously described, 65 days after treatments. In addition, boards were cut into 15-cm (N = 12) sections using a hand-held circular saw. Sections were destructively sampled using hammer and chisel to remove all live termites. The number of live termites was recorded by caste (soldier, reproductive, and workers) and board number. The presence of dead termites (dried or fungus-covered cadavers, head capsules) was noted but numbers not recorded.

## 3. Results and Discussion

This study addresses several problematic aspects of drywood termite management. The first is identification of infestation level by non-destructive sampling. This should be a requirement before deciding on an action plan for structural treatment. Second is the mechanics of delivering a control agent to attain complete coverage of the drywood termite gallery system. Third is measuring efficacy of treatments and extrapolating experimental efforts to conditions found in a structure.

The data on drywood termite activity obtained by using the Termatrac® showed a consistency that increased our confidence in the ability of that device to accurately indicate the presence of termites in 2.6-cm thick cypress boards (Table 1 & Table 5). The activity data, recorded as the number of active sections, from the control boards was consistent or increased over time in 4 of 5 boards while live termites were found in each control board at the end of the 65-day experiment (Table 1). The control board data showed that the Termatrac® was useful in detecting even small numbers of termites. For example, control board #10 consistently provided activity data at one location and only 8 termites were recovered at the end of the study (Table 1). The ability of the Termatrac® to indicate activity was verified in every one of the 14 boards where we recorded activity because on Day 65 each of those boards also provided termites using the destructive sampling technique we employed (Table 1 & Table 5). These data support the work of Mankin [14] who recorded rates of detection of stored product pests. The information listed in Table 5 is provided to illustrate relative position of activity. Of particular interest are the first two activity-reading dates taken one day apart which indicate termite activity was localized in certain sections of each board (Table 5). The difficulty in locating every single insect or area of activity was, however, highlighted by 2 boards (#2 & #6) where we obtained no Termatrac® reading but found termites by destructive sampling (Table 1 & Table 5). The rate of false negative readings, by board, was 9.5% (2 out of 21 boards) which is slightly higher than that reported by Evans [15] using subterranean termite aggregation stations. However, this should not be taken as an indictment of the device but used as a word of caution toward interpretation of inspection data. It is our opinion, based on the experience gained during this experiment, that detection of termite activity is dependent on termite movement (which we did not attempt to stimulate) and proximity of the device to the site where movement occurred. In this experiment we placed the detection device sensor (an area approximately 4 × 4-cm) within a ‘section’ of board to standardize and facilitate timely inspection within and between treatments. It seems likely that use of a technique to agitate (move) the termites and/or placing the detection device in several locations per section would have reduced the number of false negatives we obtained. These are questions that were beyond our experimental design but deserve further study because it is important to understand the probability of false negative readings with any detection device. 

Directed application of insecticide into infested structural components (local treatment) for drywood termite management is hampered in two major ways. The first, efficient verification of activity, was discussed in the preceding paragraph and second is a method to accurately locate galleries for application of treatment. This study employed a resistograph drill to ensure that voids (galleries), which were identified by a drop in resistance while drilling into the lumber, were in the path of each injection hole. Our previous, unpublished work, with drywood spot treatments always involved verification of galleries by the amount of formulation accepted by a drill hole at the time of application - for example if treatment was not ‘pushed’ from the application device into a drill hole it was assumed no gallery was intersected. This type of drill-and-treat technique necessitates drilling multiple holes over a particular length of exposed lumber, often as many as 2 holes per 5-cm of board length [16] to ensure the gallery system of a particular infestation is ‘properly treated’. The reisistograph obviated any guesswork and every drill hole treated in this study intersected a gallery. The efficacy of gallery location was verified by destructive sampling. The mean number of galleries treated by location (at the 61 & 122-cm section of each board) ranged from 1.3 to 2.8 per treatment while the mean number of resistograph holes drilled to find those galleries ranged from 3.2 to 6.8 by location, board and treatment (Table 2). It took at least 2 drill attempts with the resistograph before a gallery was intersected in most boards.

Interestingly we found, when conducting the destructive sampling, dead termites in all treatments—including the controls. However, all treatments when compared to the control provided evidence of impact on drywood termite populations using the metric of mean Termatrac® readings per board or mean number of termites per board (Table 3 & Table 4). We believe determining differences between treatments is an exercise in statistical choice and from the viewpoint of biological significance we prefer to examine the data by board as case histories without application of statistical separation (Table 5). Two reasons justify this approach. First, the variability in the number of termites removed from boards 65 days after treatment (Table 4). The mean values by treatment listed in Table 4 show that the standard deviations are equal to or exceed the mean in nearly half of the possible comparisons. This could be, largely, due to the assignment of boards to a treatment by categorization of activity levels. Yet, if one examines the number of termites recovered at the end of the trial compared to the number of active sections in that board there is no correlation between the number of live termites and the number of active sections (Table 1). Despite this lack of association, the number of active sites identified by Termatrac® do provide a measure that separate treatments because the two fipronil treatments provided at least 4 times fewer active sections per board compared to the controls and the imidacloprid treatment (Table 4). The second reason for forgoing statistical comparisons relates to the issue of the biological relevance of drywood termite local treatment efficacy standards. Determination of treatment efficacy should be decided, in part, by the biological attributes of the pest. In this report, we must consider the ability of drywood termites to produce reproductives through neoteny. Therefore, any treatment where reproductives were recovered, regardless of the numbers, must be considered less favorably than treatments that eliminated all termites from a particular board. Employing this measure of treatment efficacy we can claim that none of the treatments provided complete success because reproductives were found in at least one board from every treatment 65 days after application of insecticide. The dry and exp treatments did, however, provide evidence for complete elimination of termites from 5 of the 11 boards and low numbers as well as no reproductives in 2 additional boards (#17, #2) from the exp treatment (Table 1). It is clear from these data that the fipronil formulations were more likely to eliminate *I. synderi* infestations in 65 days using a local treatment technique compared to the imidacloprid foam formulation.

The present study was conducted as a rigorous test of these RTU products because applications were made at only two locations, 16-cm from either end of a 3-m board, and efficacy was measured after 65 days. The ‘hole’ spacing used in this study for delivery of a spot or local treatment for drywood termites should be considered extreme given the application of treatment locations used in past studies [8]. The time frame we used to evaluate treatment of naturally infested lumber should be considered short or quick compared to other studies that employ intervals from 90 days to one year [8,9,10]. Therefore we hypothesize that all of our treatments would have shown further reductions in numbers of live termites given more time… if not outright elimination. 

It is our opinion that local treatments for drywood termites are hampered by ‘incomplete’ insecticidal coverage because of the architecture of the gallery system. Table 5 shows that there was no correlation between the number of galleries in the path of the treatment and elimination of termites from a board. The gallery system of *I. snyderi* was characterized by enlarged gallery-sections connected by small diameter (2-mm) tunnels. This arrangement created bottlenecks that thwarted uniform distribution of the formulations we tested because those small galleries could have easily been blocked by fecal pellets or live termites. The fact that we only treated two locations along a 3-m board and were able to eradicate termites in 5 of 11 boards (30%) treated with the two fipronil formulations after 65 days demonstrates that the ultimate endpoint of efficacy is attainable. These data are evidence that formulation/active ingredient combinations can be developed that provide a high level of confidence in drywood termite drill-and-treat local treatments. 

## 4. Conclusions

Successful elimination of drywood termite infestations using a local treatment method depends on termites contacting the treatment after application rather than obtaining a lethal dose during application. We believe a better understanding of drywood termite behavior, improved detection/treatment techniques and patience (*i.e.*, allowing time for optimum results) will demonstrate that local treatments can provide a useful alternative to fumigation for (relatively) low-level infestations with a greater degree of certainty than currently held for this methodology. Future studies should examine insecticide formulations with markers to determine their distribution within a gallery system following application and destructive sampling over time to elucidate the role of residual activity and frequency of insect contact after application. 

## References

[1] Gay F.J., Krishna K., Weesner F.M. (1969). Species Introduced by Man. Biology of Termites.

[2] Weesner F.M., Krishna K., Weesner F.M. (1970). Termites of the Nearactic Region. Biology of Termites.

[3] Eggleton P., Abe T., Bignell D.E., Higashi M. (2000). Global Patterns of Termite Diversity. Termites: Evolution, Sociality, Symbioses, Ecology.

[4] Abe T., Cornell J.H., Hidaka T., Kawano S. (1987). Evolution of Life Types in Termites. Evolution and Coadaptation in Biotic Communities.

[5] Korb J., Lenz M. (2004). Reproductive decision-making in the termite, Cryptotermes secundus (Kalotermitidae), under variable food conditions. Behav. Ecol..

[6] Grace J.K., Woodrow R.J., Oshiro R.J. (2009). Expansive gallery systems of onepiece termites (Isoptera: Kalotermitidae). Sociobiology.

[7] Scheffrahn R.H., Robbins W.P., Busey P., Su N.-Y., Mueller R.K. (1993). Evaluation of a novel, hand-held, acoustic emissions detector to monitor termites (Isoptera: Kalotermitidae, Rhinotermitidae) in wood. J. Econ. Entomol..

[8] Lewis V.R., Haverty M.I. (1996). Evaluation of six techniques for control of the western drywood termite (Isoptera: Kalotermitidae) in structures. J. Econ. Entomol..

[9] Scheffrahn R.H., Su N.-Y., Busey P. (1997). Laboratory and field evaluations of selected chemical treatments for control of drywood termites (Isoptera: Kalotermitidae). J. Econ. Entomol..

[10] Woodrow R.J., Grace J.K., Oshiro R.J. (2006). Comparison of localized injections of spinosad and selected insecticides for the control of *Cryptotermes brevis* (Isoptera: Kalotermitidae) in naturally infested structural mesocosms. J. Econ. Entomol..

[11] Neoh K.-B., Lee C.-Y. (2011). Developmental stages and caste composition of a mature and incipient of the drywood termite, *Cryptotermes dudleyi* (Isoptera: Kalotermitidae). J. Econ. Entomol..

[12] Thoms E.M. (2000). Use of an acoustic emissions detector and intragallery injection of spinosad by pest control operators for remedial control of drywood termites (Isoptera: Kalotermitidae). Fla. Entomol..

[13] Lewis V.R., Power A.B., Haverty M.I. (2004). Surface and subsurface sensor performance in acoustically detecting the western drywood termite in naturally infested boards. For. Prod. J..

[14] Mankin R.W. (2004). Microwave radar detection of stored-product insects. J. Econ. Entomol..

[15] Evans T.A. (2002). Assessing efficacy of Termatrac™; A new microwave based technology for non-destructive detection oftermites (Isoptera). Sociobiology.

[16] Lewis V.R., Rust M. (2009). Drywood termite control. Preliminary laboratory evaluation of chemical local treatments for drywood termites. http://www.nxtbook.com/nxtbooks/naylor/CPCQ0109/index.php#/14.

